# Differences in traffic safety behaviours between male and female drivers across road categories in a developing country: A case study

**DOI:** 10.1371/journal.pone.0355724

**Published:** 2026-08-18

**Authors:** Mirjana Grdinić-Rakonjac, Vladimir Pajković

**Affiliations:** Department of Road Traffic, Faculty of Mechanical Engineering, University of Montenegro, Podgorica, Montenegro; Drexel University School of Biomedical Engineering Science and Health Systems, UNITED STATES OF AMERICA

## Abstract

Rising traffic crash rates present a significant public health challenge, particularly in developing countries where fatality rates are disproportionately high. Sex and road type are critical but underexplored factors influencing risky driving behaviours. This study aims to investigate sex-specific driving behaviours across various road types, providing insights into how males and females differ in their driving habits. Speeding, driving under the influence of alcohol, seatbelt usage and mobile phone use while driving are risky behaviours selected for analysis. Data were collected through a survey of 1309 licensed drivers. Statistical tools, namely Student’s t-test and ordinal logistic regression analysis, were used to determine gender differences while controlling for age and driving experience. Findings indicate that male drivers were generally more prone to risky behaviours such as speeding and driving under the influence of alcohol. Female drivers reported significantly higher rates (as confirmed by both the t-test (*p* = 0.007) and regression analysis (*p* = 0.029)) of extreme speeding (exceeding the speed limit by more than 30 km/h) on high-speed roads. They also demonstrated greater vigilance in ensuring the safety of themselves and their passengers by wearing seatbelts on regional (*p* = 0.003) and urban roads (*p* = 0.001). Despite previous research suggesting lower awareness of alcohol-impaired driving risks in developing countries, the findings from this study indicate that the majority of drivers exhibit a higher level of risk awareness than typically expected in such contexts. Additionally, while prior studies have shown that men are more prone to distractions, this study finds that both genders exhibit similar tendencies toward texting and using social media while driving. Findings reveal that risky behaviours are dependent on gender and context, with some results diverging from patterns established in developed countries. Tailored interventions are needed targeting men in urban areas and awareness campaigns addressing excessive speeding among women on high-speed roads.

## Introduction

The state of road safety in recent years has been a cause for concern. The EU has set ambitious targets to halve deaths and serious injuries between 2020 and 2030 and to reach close to zero by 2050 [1]. However, the current rate of progress is slow and insufficient to achieve the set goals. In addition, differences in road safety exist between developing and developed countries: although developed countries have 1.5 times the motorisation level, developing and underdeveloped countries reported an 11 times higher crash rate [2]. In Montenegro, which is a developing country, traffic crashes represent a major public health problem with a relevant mortality rate of 90 per million inhabitants, which is about twice the EU average and even about 5 times higher than in Sweden, which represent the best-performing EU country in 2022 [3]. Although the number of crashes in the world is decreasing (or stagnating in the last few years), this is not the case in Montenegro, and from 2020 on, the number is constantly rising: in 2023, the number of crashes was 15.8% higher compared to 2022, while the number of fatalities was 4.5% higher [4].

Many surveys are conducted with the aim of monitoring driver behaviours that lead to crashes and public attitudes toward road safety. However, there is a relatively small number of studies that investigate drivers’ behaviours in developing countries, and even less that examine the specifics between different groups of drivers. For example, the project named European Survey of Road Users’ Safety Attitudes (ESRA) represents a collaborative effort among research organizations and road safety institutes across 17 European countries with the main goal of gathering comparable national and international data on road user behaviours regarding road traffic risks. However, each of the countries involved are developed ones. Similar to ESRA is the project Social Attitudes to Road Traffic Risk in Europe (SARTRE), which is carried out in Europe and also includes mainly developed countries; subsequently, this research has been extended to two developing countries outside the EU: Serbia and Israel [5]. In the research conducted by Pires et al. [6], results from the 2018 ESRA survey are presented, involving 32 participating mainly developed countries from Europe, North America, Asia, Oceania, and Africa.

All these studies show that there are differences in traffic safety between countries, regardless of whether they are developed or developing. These differences can be attributed to various factors, including infrastructure quality, enforcement of traffic laws, vehicle safety standards, etc. However, these differences become more pronounced when a developed country is compared to one that is still developing, where disparities in resources, regulatory frameworks, and safety cultures further amplify variations in road safety outcomes. Sheykhfard et al. [2] investigated how do driving behaviour and attitudes toward road safety vary between developed (the Netherlands) and developing (Iran) countries. Their findings indicate that drivers in developing regions exhibit a higher tendency for traffic violations, aggressive infractions, and risky driving habits, whereas those in developed regions demonstrate safer behaviour, fewer infractions, and greater adherence to traffic regulations. Furthermore, Sheykhfard et al. [2] found that attitudes toward road safety in developing regions were significantly less secure, that is, characterised by a higher propensity to tolerate or rationalize traffic rule violations and a lower willingness to comply with them consistently, which in turn contributed to a higher prevalence of risky behaviours. These differences have been attributed to the fact that developed countries invest in public awareness campaigns, driver education programs, and initiatives to promote safe driving behaviour, whereas developing countries often lack these actions, leading to a higher incidence of risky behaviours and a limited understanding of the importance of obeying traffic rules [2].

Studies conducted in developed countries provide valuable insights, however, their findings may not accurately reflect the situation in developing countries. Moreover, even when similar behaviour patterns are observed, the same strategies may not yield equally effective results due to differences in local conditions and contextual factors. It is necessary to examine how driver behaviour specifically affects road safety in each country, in order to identify key risk factors and establish a foundation for targeted preventive measures. So, the primary goal of this study is to identify specific driver behaviours that contribute to the occurrence of traffic crashes in a developing country context, with Montenegro serving as the empirical case study. Through a detailed analysis of the local situation, the study aims to provide a comprehensive understanding of driver behaviours that negatively impact traffic safety, which is essential for the development of targeted preventive measures. Furthermore, various studies [7,8] have shown that road users behaviour is influenced by multiple factors such as sex, age, experience, etc. This was a motivation for the authors to expand the scope of this study to also examine sex differences in driving behaviours in order to identify specific groups of drivers and the particular behaviours that pose a risk in traffic.

Road type (e.g., highways, rural roads, urban streets) plays a critical role in shaping road safety outcomes. Variations in geometric design, traffic conditions, and environmental factors directly impact accident frequencies and driver behaviour [9]. Existing research has primarily examined how geometric design influences driving behaviour across different road types [10,11], how traffic conditions affect road safety [12], and the role of road maintenance in mitigating risks [13]. Additionally, environmental factors, such as weather and visibility, have been explored in relation to their impact on driver behaviour [14]. Despite these extensive investigations, a critical gap remains in understanding how driver characteristics, particularly sex differences, influence behaviour across various road categories. While prior studies have highlighted general trends in driver behaviour, few have specifically analysed how male and female drivers adapt their driving styles in response to different road types. To fulfil this gap in the literature, present study examines variations in driver behaviour across different road types. By incorporating sex-specific analyses, this research aims to provide a more nuanced understanding of how behaviour is shaped by road environments, ultimately informing policies and infrastructure improvements that enhance overall traffic safety.

Some previous studies examining differences between male and female drivers in terms of crash involvement, behaviours, road type and country development are presented in the next section, after which the methodology of the research is presented. Results and discussion are given next, following with summarised conclusions.

## Literature review

When it comes to traffic safety, great efforts are made to show the psychological understanding of driver behaviour and perceived risk. The opinions and habits of different groups of road users are a source of relevant information as they reveal characteristics related to mobility, their relationships with other users, their experiences, and their safety concerns. Existing literature highlights significant differences in traffic crash involvement [15–19] and also in road safety behaviour between men and women [20,21], including differences in risky driving [2,16], traffic violations [6], risk-taking behaviour [16,21,22], perceptions and attitudes towards road safety [2,23], etc. Researchers suggest that, for each country, gender-specific interventions and educational programs could be beneficial in reducing road traffic crashes [7]. To provide insights into the previously identified differences between male and female drivers, the following section presents a review of relevant studies. The literature review is structured to highlight these differences across four categories: involvement and injuries in road crashes, attitudes toward traffic safety and risky driving behaviours, related to road type and also related to country development.

### Differences in road crash involvement and injury outcomes

McCarty & Kim [22] explored the influence of sex on road crash rates in England and concluded that the higher prevalence of risky behaviours among males resulted in their greater involvement in road crashes compared to female drivers. In Malaysia, men are also identified as a riskier group of drivers [15]. The authors noted that about 80% of road deaths among individuals are men. Similar findings have been reported in Mahmood et al.’s [16] research conducted among Pakistani drivers. It was shown that, because of their greater likelihood of engaging in hazardous driving practices, the casualty rate for male drivers is more than triple that of female drivers. On the contrary, research on Spanish drivers showed that women tend to experience more severe health consequences compared to men when involved in car crash, despite men being involved in more crashes overall [17]. Similarly, the study conducted by Atubi [18] in Lagos showed that, despite being less frequently involved in crashes, women tend to suffer more severe injuries when they are involved in crashes. Atubi [18] extended their research to investigate crashes involving different types of vehicles. Additionally, their findings indicate that men are more frequently involved in road traffic crashes compared to women across various vehicle types, including small cars, minibuses, and four-wheel drives. The same research also shows that a notable difference exists in the types of vehicles driven by men and women, indicating that men are more likely to drive larger vehicles such as minibuses and four-wheel drives, while women are more likely to drive smaller cars. This difference in vehicle types can influence the severity of injuries in the event of a crash due to the varying levels of protection provided by different vehicle sizes and types. Pires et al. [6] present summarized findings from the ESRA project conducted in 2018 across 32 developed countries. Their findings show that in every country studied, a majority of drivers involved in traffic crashes were male. In contrast, while female drivers reported more errors, they were involved in fewer severe traffic crashes. Also, male drivers in New South Wales, Australia, were less likely to be involved in crashes resulting in hospitalization compared to female drivers [19]. In their research, Cullen et al. [19] conducted a 13-year study in order to explore sex differences in crashes and crash-related injuries among young Australian drivers. They found that male drivers had higher rates of any crash, single vehicle crash, crash on streets with a speed limit of 80 km/h or above, crash in wet conditions, and crash in the dark, respectively.

### Differences in attitudes and risky driving behaviours

It is a fact that there are notable differences in attitudes toward road safety between sexes. Women generally show a greater respect for road safety norms [2], a higher level of compliance with traffic rules and a more cautious approach to driving [2,6]. In addition, Atombo & Wu [23] claimed that both male and female drivers’ positive driving behaviours are influenced by perceived internal requirements and traffic system functionality. Male drivers tend to be less concerned by road safety [24] and are more likely than female drivers to lose license points due to traffic violations [9]. On the other hand, while female drivers are generally more compliant with traffic rules and regulations [16], they may exhibit more driving anger than men, largely due to their heightened concern for road safety and increased susceptibility to distraction caused by the inappropriate driving behaviours of others [25]. However, they are more likely to remain calm and avoid showing signs of rage while driving [16] and tend to express anger in more subtle ways, such as swearing or whispering insults [25]. Studies show that male drivers tend to take more chances and are more prone to aggressive driving [2,21] and using the vehicle to express anger [25], especially during their teenage years [23]. Hashmi et al. [25] show that males tend to react more angrily and aggressively while driving, especially in traffic jams and seem to exhibit more explicit and directly observable aggressive behaviours while driving, like obscene gestures [16]. In addition, Barr et al. [26] found that male teen drivers report to engage in risky behaviours more frequently than females, despite often perceiving themselves as safe drivers. Female drivers are generally more compliant with traffic rules and regulations and males are less bothered with road safety [16]. Studies show that there is a greater discordance between perceived and actual risk in male drivers than in females [6]. Women demonstrated a heightened awareness of the risks associated with reckless driving [6], emphasized the need for stricter pre-licensing education, and showed greater support for young driver restrictions, including speed limits and night-time driving bans, reflecting their higher perception of risk associated with these behaviours [6,27].

Generally, number of studies confirm that drivers sex significantly predicts risky behaviours, with male drivers more likely to engage in such behaviours compared to female drivers [6,21]. A greater number of fines and a lower level of motivation to comply with traffic laws are recorded for male drivers [25]. Males are more likely to report being cited for moving violations and engage in personal distractions such as using cell phones, smoking, and eating while driving as was shown in [26]. Also, using a mobile phone while driving and excessive speeding were significantly higher in male drivers as compared to females [28]. Moreover, several studies have shown that males are also more likely to engage in behaviours such as speeding and driving under the influence of alcohol [2,16,18,22]. In addition, male drivers were less likely to use seatbelts themselves and less likely to encourage passengers to use seatbelts, compared to females [26]. In contrast to what was mentioned, female drivers tend to be more cautious and compliant with traffic laws. Generally, women exhibit relatively safer driving behaviours. Lucas et al. [29] found that female drivers tend to drive under speed limits more in different situations, such as rush hour, local roads, and highways. However, certain violations, such as running red lights and using the emergency lane, are more common among women compared to male drivers [15]. Sex seems to be influential for non-drivers as well. For example, Dinh et al. [20] analysed self-reported pedestrian behaviour in China and concluded that male road users reported less-safe behaviours.

The greater overall driving safety observed among women compared to men can be attributed to several key factors. Hashmi et al. [25] suggest that male drivers tend to perceive traffic environments as less emotionally demanding and often overestimate their driving skills, which reduces their likelihood of adhering to safety regulations. This aligns with prior research indicating that males, especially young men, are more prone to traffic violations and crashes [30,31]. In contrast, women are more concerned about safety in traffic, which fosters more positive driving behaviours [23] and results in safer driving practices, especially when have children on board [16].

### Differences related to road type

There is strong evidence in the literature that driver behaviour is significantly influenced by the road itself, including its characteristics and design, which affect driving conditions, speed adaptation, and manoeuvring patterns [9–11,32]. Road category plays a critical role in traffic safety, impacting both accident frequency and severity. Empirical studies indicate that crashes are more frequent and severe on rural roads compared to urban environments, while higher-standard roads such as motorways, generally provide safer conditions due to superior design and controlled access [32–34]. The interaction between roadway features and driver behaviour has profound implications for overall traffic safety. A key contributing factor of these trends is risk perception, which varies across different road environments [9,14]. For instance, on highways, drivers often perceive high speeds as less risky due to elevated speed limits and reduced visual complexity, fostering a false sense of security that may encourage riskier behaviours [9,33].

Sheykhfard et al. [14] confirm that road category plays a crucial role in shaping driver behaviour, particularly in speed selection and risk perception. They showed that drivers reduce speed in residential areas compared to rural or commercial roads. Similarly, McCarty & Kim [22] report that speeding and neglecting traffic regulations are consistent across different road types, particularly in environments with higher speed limits and fewer traffic control measures. Their study further highlights that male drivers exhibit a greater propensity for risky behaviours than female drivers. Coropulis et al. [10] explores the influence of road geometry on driver behaviour and safety and found that road categories impact speed and risk levels, abstracting rural roads as the most dangerous due to limited lane width, lack of physical separation between opposing traffic, and challenging curves. In their work, findings indicate that road geometry significantly impacts driver speed choices. In addition, they suggest that road design significantly influences speed selection, with rural roads also exhibiting higher instances of distracted driving, likely due to perceived lower enforcement and reduced risk awareness. Additionally, rural road conditions contribute to increased engagement in high-risk behaviours such as driving under the influence and seatbelt non-compliance, exacerbated by misperceptions of safety fostered by the road environment [10].

### Differences related to country development

While attitudes toward traffic safety and driving behaviours vary within a single country, the differences are even more pronounced when comparing different countries, particularly when contrasting developed and developing countries [35]. These disparities may stem from variations in economic, infrastructural differences, law enforcement effectiveness, cultural norms or policy implementation [7,36,37]. Additionally, socioeconomic factors play a critical role, as economic constraints and limited access to safe transportation can contribute to riskier driving behaviours [7].

Lund & Rundmo [7] examined cross-cultural differences in risk perception between a developed (Norway) and a developing (Ghana) country, with a focus on traffic safety. Their findings revealed significant variations in attitudes, risk perception, and driving behaviour. For example, hazardous traffic conditions and fewer formalized driving regulations characterised for developing country contribute to greater risk perception, while structured traffic laws and safety campaigns in developed countries result in lower risk perception. Additionally, while male drivers in the developed country perceived risks as lower than female drivers, no significant gender differences in risk perception were observed in the developing country. Notably, despite reporting higher risk perception, drivers in the developing country experienced more crashes, suggesting that increased awareness of risk does not necessarily translate into safer driving behaviour. Schlembach et al. [5] investigated traffic safety culture and alcohol use in European countries based on the SARTRE 4 study. Their findings highlight significant differences between developed and developing countries in terms of risk awareness and legal consciousness regarding drinking and driving. In developed countries, drivers demonstrate greater awareness of the dangers associated with alcohol consumption while driving and also are more aware of the legal consequences, which influences their behaviour. In contrast, in developing countries, risk awareness tends to be lower, likely due to weaker law enforcement and reduced exposure to public safety campaigns. Additionally, the study identifies notable sex differences in driving behaviours and risk perception. Men, especially in developing countries, exhibit higher rates of risky driving behaviours and lower risk perception, making them more susceptible to alcohol-related traffic violations. In contrast, women generally have more cautious attitudes and higher safety awareness, regardless of the country’s development level. Granié et al. [38] showed that sex has a stronger influence on individual risk behaviours than broader societal norms. They found that personal acceptance and self-reported behaviours vary significantly across cultures. Their findings indicate that gender differences in risk behaviours are more pronounced in developed countries than in developing ones. For instance, self-reported drinking and driving rates show a larger gender gap in developed countries, with men reporting significantly higher rates of this behaviours than women. Similarly, seatbelt use in developed countries is higher among women than men, whereas in developing countries, overall seatbelt usage is lower, but the gender disparity is smaller. Speeding is consistently more prevalent among men than women everywhere, but the sex difference is larger in developed countries, where higher driving speeds and extensive highway networks contribute to riskier driving patterns. Unlike other risky behaviours, Granié et al. [38] found that gender differences in mobile phone use while driving remain consistent across all country development levels, with men being more likely to engage in this behaviour in all regions.

All of these presented findings highlight significant differences in risk perception between developed and developing countries, emphasizing the influence of cultural and environmental factors on safety attitudes and behaviours. In some countries, sex disparities in risk perception and driving behaviours are more pronounced, so addressing these disparities requires tailored interventions, including stronger policy enforcement, infrastructure development, and targeted public awareness campaigns to improve overall road safety outcomes [7]. In developing countries, higher risk perception does not necessarily translate into safer behaviours, suggesting that external constraints, such as poor enforcement of traffic laws and inadequate infrastructure, play a crucial role [37]. This paper presents a comprehensive analysis of data collected from questionnaires administered across the Montenegro. These data are further analysed to identify distinct behavioural patterns and risk factors associated with male and female drivers. The evidence-based findings aim to assist policymakers in developing gender-responsive targeted interventions to effectively improve overall traffic safety in the country.

## Materials and methods

### Study design and procedure

As previously stated, to increase traffic safety, it is necessary to examine factors that contribute to crashes and analyse risky behaviours that lead to incidents. In a developing country such is Montenegro, there are often no relevant and reliable measures on driver behaviour in traffic, so data can be gathered through questionnaires. In the literature, behavioural data collected through surveys is known as self-reported behaviour [26,39].

The Montenegrin version of questionnaire was designed following the ESRA and SARTRE methodologies and includes section on sociodemographic and personal characteristics (age, gender, occupation), section on information related to driving (experience, driving frequency) and, includes sections on behaviours related to self-reported driving behaviours (related to speeding, driving under the influence of alcohol, seat belt use, and mobile phone use while driving on different road types, namely main, regional and urban roads). The survey was conducted anonymously, ensuring that no personal information was collected from respondents. Each respondent indicated only the frequency of driving-behaviours using the 6-point Likert scale ranging from never, rarely, sometimes, often, very often, to always. This approach provides unbiased and authentic responses, as participants were not concerned about their identities being revealed. The questionnaire was available in both electronic (google forms) and paper formats. The survey was conducted from June to November 2019, when the electronic version was distributed via social networks, while paper forms were distributed to insurance company branches in all of 25 Montenegrin cities (Andrijevica, Bar, Berane, Bijelo Polje, Budva, Cetinje, Danilovgrad, Gusinje, Herceg Novi, Kolašin, Kotor, Mojkovac, Nikšić, Petnjica, Plav, Pljevlja, Plužine, Podgorica, Rožaje, Tivat, Tuzi, Ulcinj, Šavnik, Žabljak and Zeta). The minimum sample size was determined using a standard proportion-based method (Cochran’s formula) with a 95% confidence level and a 3% margin of error, resulting in a minimum of approximately 1,067 participants from the target population of licensed adult drivers.

### Ethics statement

Research aimed to assess the frequency of specific driving behaviours through a questionnaire distributed both, online and in paper form at insurance companies. At the beginning of the questionnaire, participants were provided with a detailed explanation of the study, including its purpose, the institution conducting the research, and an explicit statement that participation was entirely anonymous. No personal or identifiable information was collected. The study did not involve sensitive personal data or vulnerable populations. Regarding participant consent, informed consent was implicitly obtained. Participants were clearly informed that their responses would be anonymous and used solely for research purposes. By voluntarily completing the questionnaire, they confirmed their willingness to participate in the study. Since no minors were included, parental or guardian consent was not applicable. The study was approved by the Institutional Review Board (IRB) of the Faculty of Mechanical Engineering, University of Montenegro (protocol no. 1-589/1).

### Statistical analysis

The analysis of self-reported behaviour related to speeding, driving under the influence of alcohol, seat belt use, and mobile phone use while driving on main, regional and urban roads was conducted according to sex. The descriptive statistic was conducted to capture insight into the frequency of selected behaviours. In addition, positive behaviour was assigned a score of 6, whereas a negative behaviour was assigned a score of 1, indicating a higher mean reveals safer driving behaviour. After the initial descriptive analyses were conducted, overall mean scores related to the above mentioned behaviours are calculated and Student’s t-test was used first to determine if significant differences exist between male and female drivers across various road types. This test (p < 0.05) provides an initial indication of whether gender plays a role in influencing specific driving behaviours. Subsequently, an ordinal logistic regression model was employed to examine the relationship between gender and self-reported driving behaviours. The dependent (outcome) variables were the responses to driving behaviour questions, measured on an ordinal scale (responses ranging from 1 = “always” to 6 = “never”), representing the likelihood of engaging in specific driving behaviours. The independent (predictor) variable was sex (male/female), a categorical predictor used to assess its impact on the probability of selecting different response categories. Control variables were age and driving experience, since existing literature highlights these factors to be significant in traffic crash involvement and also in road safety behaviour [16,17,20,22,28,30]. Given that the distances between response categories are assumed to be unequal and are unknown [40], the ordinal logit model was found to be more appropriate [41]. This approach provides a more precise evaluation of how gender affects response probabilities, providing deeper insights into behavioural patterns beyond simple mean comparisons. The analysis was conducted using XLSTAT (version 26.2.2.0) within Microsoft Excel™ 2016.

## Results

The crucial key to the development of gender-specific interventions to improve traffic safety lies in understanding and addressing the different behaviours, attitudes, and risk factors associated with male and female drivers. Therefore, comprehensive data on driving behaviours was collected to gain insight into sex-specific unsafe driving practices. After some drop outs, the final sample was composed of 1309 drivers with 45.64% of male respondents and 54.36% of female respondents mostly employed, who drive everyday (Table 1). Statistically, no significant difference is recorded for the variable “sex” in matters related to age, employment status, frequency of driving, or years with a driving license (with the significance level 0.05). The survey’s accuracy was determined using the Cronbach’s alpha reliability coefficient, which is 0.829. Higher values of this coefficient, usually above 0.7, indicate a greater level of reliability for the scale [42–44].

**Table 1:** Descriptive statistic of the survey sample.

Variables		Male (%)	Female (%)
Age	17–24	25.96	22.53
	25–34	34.99	32.84
	35–44	16.93	22.53
	45–54	11.96	11.58
	55–64	7.00	6.95
	65+	3.16	3.58
Employment status	Student	20.82	17.48
	Unemployed	26.77	32.62
	Employed	52.40	49.89
Frequency of driving	Everyday	75.85	76.11
	1–3 times per week	15.26	14.38
	1–3 times per month	4.33	4.86
	Less than once per month	4.56	4.65
Years with driving license	0–5	28.83	27.03
	6–10	21.05	20.50
	11–20	30.89	29.05
	21–30	10.07	11.49
	31–40	7.55	9.46
	40+	1.60	2.48
Total sample		45.64	54.36

### Frequency of risky behaviours

Several behaviours were selected to analyse: driving over the speed limit, driving under the influence of alcohol, wearing the seatbelt while driving, and usage of mobile phone while driving. Results are presented in relation to sex and type of road. The descriptive results, which highlighted several notable behavioural driving differences between male and female drivers, are presented, following analysis using t-test and ordinal logit model. The frequency of self-reported speeding in the total sample is shown in Table 2.

**Table 2:** Frequency of responses in %.

Survey questions (%)	Sex	Never	Rarely	Sometimes	Often	Very often	Always	Mean	*P(T<=t)*
**Driving above the speed limit**									
How often do you drive over the speed limit?									
[On main roads]	Males	18.25	28.57	32.25	14.85	2.97	3.11	4.35	*0.063*
	Females	23.26	27.91	25.91	9.14	7.81	5.98	4.32	
[On regional roads]	Males	27.34	33.47	24.27	10.32	2.09	2.51	4.66	*0.364*
	Females	35.93	26.32	22.02	7.45	4.30	3.97	4.70	
[On local/urban roads]	Males	42.04	29.61	18.99	5.87	1.26	2.23	4.99	*0.388*
	Females	46.33	29.00	14.50	4.83	2.50	2.83	5.03	
Do you exceed the prescribed speed on roads with higher permitted speeds (60-80 km/h)									
[By more than 30 km/h]	Males	43.08	30.49	16.22	6.99	1.82	1.40	5.02	*0.007*
	Females	45.62	24.46	16.36	7.27	3.80	2.48	4.93	
[By less than 30 km/h]	Males	21.16	27.93	23.70	16.64	5.22	5.36	4.27	*0.214*
	Females	27.91	28.24	17.73	10.51	6.73	8.87	4.33	
Do you exceed the prescribed speed on roads with lower permitted speeds (50-60 km/h)?									
[By more than 20 km/h]	Males	32.59	30.35	22.80	9.65	2.38	2.24	4.74	*0.049*
	Females	41.45	25.49	20.39	8.06	2.80	1.81	4.89	
[By less than 20 km/h]	Males	21.46	24.26	26.93	16.41	6.31	4.63	4.24	*0.112*
	Females	25.99	25.99	24.34	9.54	5.76	8.39	4.32	
Do you exceed the prescribed speed on local/urban roads?									
[By more than 10 km/h]	Males	36.95	28.35	23.27	6.63	2.40	2.40	4.84	*0.187*
	Females	46.60	23.38	18.08	6.97	2.99	1.99	4.98	
[By less than 10 km/h]	Males	27.43	26.30	21.94	15.05	4.36	4.92	4.43	*0.042*
	Females	34.34	26.77	16.16	8.59	5.39	8.75	4.50	
**Driving under the influence of alcohol**									
Do you drive under the influence of alcohol?									
[On main roads]	Males	81.34	11.80	4.66	1.51	0.41	0.27	5.71	*0.222*
	Females	84.46	10.25	3.80	0.33	0.66	0.50	5.76	
[On regional roads]	Males	74.62	14.95	6.31	2.74	0.69	0.69	5.58	*0.104*
	Females	78.51	10.41	6.78	2.64	1.16	0.50	5.61	
[On local/urban roads]	Males	72.29	11.39	8.64	4.25	2.06	1.37	5.43	*0.006*
	Females	77.48	6.79	7.12	4.47	3.31	0.83	5.48	
**Wearing the seatbelt**									
How often do you wear your seatbelt while driving?									
[On main roads]	Males	5.76	6.72	10.70	10.15	7.54	59.12	4.84	*0.109*
	Females	7.26	6.11	10.73	9.24	6.93	59.74	4.82	
[On regional roads]	Males	8.73	5.87	14.19	10.91	7.23	53.07	4.61	*0.001*
	Females	10.45	10.28	12.60	7.96	6.80	51.91	4.46	
[On local/urban roads]	Males	12.50	7.55	13.19	9.62	9.48	47.66	4.39	*0.000*
	Females	19.01	9.09	10.58	9.42	5.79	46.12	4.12	
How often your front passenger is wearing a seatbelt while driving?									
[On main roads]	Males	7.96	8.92	13.85	9.88	14.13	45.27	4.49	*0.452*
	Females	9.95	6.80	13.43	9.45	10.95	49.42	4.53	
[On regional roads]	Males	7.54	10.70	16.19	11.66	14.54	39.37	4.33	*0.232*
	Females	11.78	9.00	15.38	10.64	11.78	41.41	4.26	
[On local/urban roads]	Males	11.00	11.97	18.57	9.22	15.13	34.11	4.08	*0.029*
	Females	17.36	9.42	16.69	10.74	8.93	36.86	3.95	
Do you use the child resistant system for children under the age of 5?									
[On main roads]	Males	17.70	5.29	10.34	8.28	10.57	47.82	4.32	*0.132*
	Females	13.70	4.57	12.26	6.73	5.05	57.69	4.58	
[On regional roads]	Males	18.16	6.90	10.34	7.36	12.41	44.83	4.23	*0.090*
	Females	14.49	5.07	11.35	7.25	6.28	55.56	4.52	
[On local/urban roads]	Males	16.39	8.20	12.88	8.67	12.65	41.22	4.17	*0.155*
	Females	14.76	5.95	11.43	8.10	6.19	53.57	4.46	
**Using mobile phone while driving**									
Do you use a hands-free device to talk to a mobile phone while driving?									
[On main or regional roads]	Males	67.04	10.85	8.48	5.29	1.95	6.40	1.83	*0.086*
	Females	67.60	10.25	8.93	4.13	2.98	6.12	1.83	
[On local/urban roads]	Males	69.44	10.00	8.61	4.17	2.78	5.00	1.76	*0.015*
	Females	72.07	10.74	6.61	3.97	1.98	4.63	1.67	
Do you use a mobile phone while driving to write messages and visit social networks?									
[On main or regional roads]	Males	60.31	18.66	11.28	5.71	2.37	1.67	5.24	*0.642*
	Females	60.40	18.48	10.73	6.27	1.82	2.31	5.22	
[On local/urban roads]	Males	57.36	18.06	10.69	6.11	4.03	3.75	5.07	*0.131*
	Females	59.77	13.25	11.26	7.95	4.30	3.48	5.06	

It can be observed that drivers are generally more careful on city streets and tend to drive above the speed limit, mainly on main roads. Additionally, the analysis of speeding on different types of roads shows notable sex differences. It appears that female drivers are more considerate about speeding compared to male drivers, as a larger percent of females reported never driving over the speed limit on main roads. To assess the severity of speeding based on gender, drivers were asked about the frequency and extent of this behaviour. Results also indicate that a higher proportion of females always exceeding the speed limit, indicating greater polarization in self-reported speeding behaviour among female drivers. On roads with higher permitted speeds (60–80 km/h), females (M = 4.93, SD = 1.28) were significantly (*p* = 0.007) more likely to exceed the speed limit by more than 30 km/h compared to males (M = 5.02, SD = 1.12). This finding suggests that woman may engage in riskier speeding behaviours on higher-speed roads. However, when the excess speed was less than 30 km/h, no significant gender difference was observed (*p* = 0.214). Similarly, on roads with lower permitted speeds (50–60 km/h), males (M = 4.74, SD = 1.20) were significantly more likely to exceed the speed limit by more than 20 km/h compared to females (M = 4.89, SD = 1.21, *p* = 0.049). However, for lesser speeding infractions (less than 20 km/h over the limit), the sex difference was not significant (*p* = 0.112). In addition, 46.6% of women reported never exceeding the speed limit by more than 10 km/h on city streets, compared to only 37% of men. Significant sex differences were observed for speeding by less than 10 km/h over the limit (*p* = 0.042) suggesting that speeding is more likely to males. However, not for speeding by more than 10 km/h (*p* = 0.187). These findings indicate a tendency for males (M = 4.43, SD = 1.40) to engage in more extreme speeding behaviours than females (M = 4.50, SD = 1.58) on roads with lower speed limits.

Driving under the influence of alcohol was assessed with the item “Do you drive under the influence of alcohol?”. The analysis of the data on driving under the influence of alcohol presented reveals several important insights into the behaviours of males and females. Analysis shows that drivers are largely careful with driving after consuming alcohol, with approx. three-quarters of the respondents claim that they do not practice this behaviour. Once again, women have proven to be safer drivers regardless of the type of road, consistently reporting slightly higher percentages of never driving under the influence on main roads (84.5% of females compared to 81.3% of males), on regional roads (78.5% of females compared to 74.6% of males), and on urban roads (77.5% of females compared to 72.3% of males). This suggests that females are generally less likely to engage in such risky behaviour with a marginal significant difference only when observed on city streets, with males (M = 5.43, SD = 1.09) reporting higher incidences of driving under the influence (*p* = 0.006) compared to females (M = 5.48, SD = 1.11). This finding is critical as it highlights a higher risk behaviour among males in environments where pedestrian traffic is likely higher, increasing the potential for crashes and fatalities.

Seatbelt usage showed significant sex differences in various contexts. The research indicates that drivers tend to be more relaxed when driving on urban streets, which is also evidenced by the reduced frequency of seat belt usage on this type of road. While self-reported seatbelt usage was not significantly different between sexes on main roads (*p* = 0.109), males reported significantly higher usage rates on regional roads (M = 4.61, SD = 1.73, *p* = 0.001) and urban roads (M = 4.39, SD = 1.85, *p* = 0.000) in contrast to female drivers (M = 4.46, SD = 1.85 on regional, and M = 4.12, SD = 2.02 on urban roads). When considering front passengers, males reported that their passengers tend to wear seat belts less frequently than their female counterparts across all usage categories. However, significantly higher rates of ensuring their front passengers wore seatbelts (*p* = 0.029) was recorded only on urban roads where males (M = 4.08, SD = 1.78) reported higher rates compared to females (M = 3.95, SD = 1.91). This disparity underlines the need for targeted interventions to enhance seat belt usage, particularly among female drivers and passengers. Finally, no significant sex difference was observed related to the usage of child resistant system for children under the age of five, although females consistently report higher child seat usage rates for children, compared to males, on each road type. It is not a surprise since cultural and social norms influence females to take on more responsibility for the safety measures of children [16,30]. Higher frequencies of this behaviour are reported on main roads compared to regional and urban roads for both males and females. However, it is still concerning that a significant proportion of drivers, particularly men but also women, never or rarely transport children in the child seat.

The use of mobile phones while driving demonstrated significant sex differences related to the usage of hands-free devices on urban roads, where females (M = 1.67, SD = 1.32) reported significantly lower (*p* = 0.015) usage of hands-free devices compared to males (M = 1.76, SD = 1.39). This suggests that males might be more inclined to use technology that allows for hands-free communication while driving, potentially mitigating the risks associated with handheld phone use. However, when writing messages or visiting social networks while driving in matter, both males and females are equally likely to engage in texting and visiting social networks while driving and no significant differences were found between genders on both main and regional roads (*p* = 0.642) and urban roads (*p* = 0.131). It can be observed that this behaviour is more common on city streets in contrast to main and regional roads. The reason might be the nature of traffic flow on urban roads with smaller permitted speeds and a large number of stops on traffic lights, compared to main and regional roads where high speed continuous driving is common.

### Results of regression analysis

This study aimed to examine sex disparities in speeding, driving under the influence of alcohol, seatbelt usage, child-resistant system, and mobile phone use while driving by collecting frequencies of answers of these self-reported behaviours. Since answers were in the form of ordinal variables where the frequency is reported from never to always, ordinal logit model was used to calculate cumulative probability of responding. Sex was set as explanatory variable and if probability Pr > Chi² is less than a significance threshold which has been set at 0.05 then the contribution of the sex to the adjustment of the model is significant. Also, the absolute value of a coefficient indicates relationship of the explanatory variable (in this case independent variable is gender coded with 0 for males and 1 for females) to response variable (in this case dependent variables refer to likelihood of a certain self-reported behaviour). Relying on this, positive obtained coefficient indicates higher probability of examined behaviour for women and negative coefficient indicates higher probability for men. Additionally, incorporating control variables such as age and driving experience is essential when examining the influence of sex on driver behaviour. Age and experience are significant determinants of driving behaviour, and their control ensures that observed gender differences are not attributable to these variables. All results are presented in Table 3.

**Table 3:** Gender-related differences of selected behaviours.

Survey questions	Mean	SD	Value	Wald Chi-Square	Pr > Chi²	Wald bound (95%)
**Driving above the speed limit**						
How often do you drive over the speed limit?						
[On main roads]	3.36	1.32	−0.038	1.336	0.248	[-0.103, 0.027]
[On regional roads]	3.72	1.28	−0.016	0.233	0.630	[-0.081, 0.049]
[On local/urban roads]	4.05	1.20	−0.022	0.397	0.529	[-0.088, 0.045]
Do you exceed the prescribed speed on roads with higher permitted speeds (60–80 km/h)						
[By more than 30 km/h]	4.05	1.18	−0.075	4.788	0.029	[-0.142, -0.008]
[By less than 30 km/h]	3.40	1.45	−0.002	0.002	0.961	[-0.066, 0.063]
Do you exceed the prescribed speed on roads with lower permitted speeds (50–60 km/h)?						
[By more than 20 km/h]	3.94	1.16	−0.064	3.656	0.056	[-0.130, 0.002]
[By less than 20 km/h]	3.41	1.41	−0.021	0.400	0.527	[-0.085, 0.044]
Do you exceed the prescribed speed on local/urban roads?						
[By more than 10 km/h]	4.00	1.19	−0.021	0.391	0.532	[-0.088, 0.045]
[By less than 10 km/h]	3.57	1.47	−0.026	0.630	0.427	[-0.091, 0.038]
**Driving under the influence of alcohol**						
Do you drive under the influence of alcohol?						
[On main roads]	4.73	0.72	−0.051	1.076	0.300	[-0.147, 0.045]
[On regional roads]	4.60	0.88	−0.042	0.946	0.331	[-0.126, 0.043]
[On local/urban roads]	4.51	1.03	−0.065	2.334	0.127	[-0.149, 0.018]
**Wearing the seatbelt**						
How often do you wear your seatbelt while driving?						
[On main roads]	4.98	1.59	0.051	1.828	0.176	[-0.023, 0.124]
[On regional roads]	4.63	1.77	0.106	9.115	0.003	[0.037, 0.176]
[On local/urban roads]	4.38	1.94	0.118	11.594	0.001	[0.050, 0.186]
How often your front passenger is wearing a seatbelt while driving?						
[On main roads]	4.60	1.70	0.011	0.107	0.743	[-0.056, 0.078]
[On regional roads]	4.38	1.72	0.027	0.654	0.419	[-0.038, 0.092]
[On local/urban roads]	4.14	1.82	0.065	3.889	0.049	[-0.000, 0.130]
Do you use the child resistant system for children under the age of 5?						
[On main roads]	5.22	2.01	−0.001	0.000	0.987	[-0.066, 0.065]
[On regional roads]	5.16	2.04	−0.006	0.028	0.867	[-0.070, 0.059]
[On local/urban roads]	5.04	2.04	−0.011	0.119	0.730	[-0.076, 0.053]
**Using mobile phone while driving**						
Do you use a hands-free device to talk to a mobile phone while driving?						
[On main or regional roads]	1.93	1.51	0.077	4.348	0.037	[0.005, 0.149]
[On local/urban roads]	1.82	1.43	0.106	7.583	0.006	[0.030, 0.181]
Do you use a mobile phone while driving to write messages and visit social networks?						
[On main or regional roads]	4.33	1.07	−0.002	0.002	0.964	[-0.074, 0.071]
[On local/urban roads]	4.24	1.22	−0.036	0.961	0.327	[-0.109, 0.036]

The effect of sex on speeding varies by road type. Negative values of coefficients calculated for speeding indicate to a slight negative relationship between gender and speeding, suggesting that the probability of speeding is higher for women. However, these effects are not statistically significant since *p* < 0.05, except for speeding by more than 30 km/h on roads with higher permitted speeds (60–80 km/h) where *p* = 0.029. The negative coefficient for gender across all road types suggests that women (coded as 1) are more likely to report driving under the influence of alcohol compared to men (coded as 0). Therefore, women are more prone to report higher frequencies of driving under the influence, indicating less safe behaviour in this particular analysis, though not statistically confirmed. Sex has a statistically significant effect on wearing a seatbelt on regional and urban roads, with a *p* = 0.003 and *p* = 0.001, respectively. Positive coefficients (0.106 and 0.118) suggests that the probability of wearing the seatbelt is higher for females compared to the males. The confidence intervals do not include zero, which further confirms the significance of the effect. Results show that there is a statistically significant effect of sex on the usage of hands-free device to talk to a mobile phone while driving on all roads (p < 0.5), with males exhibiting a higher likelihood of usage compared to females. However, using a mobile phone for writing a messages or using social networks on main, regional and urban roads did not show a statistically significant effect.

All of these results, discussed in the next section, can be useful for decision makers developing targeted traffic policy and traffic safety campaigns.

## Discussion

The findings of this study reveal notable differences in several aspects of driving behaviour among drivers in a developing country, particularly in relation to speeding and driving under the influence of alcohol, as well as the use of seatbelt, child restraint systems and mobile phone while driving. These behaviours also vary by sex and across different types of roads. It is important to note that some inconsistencies emerged between the results of the descriptive analyses and those obtained from the ordinal logistic regression models. This was no surprise since the two approaches capture different statistical aspects of the data. Descriptive tests provide unadjusted comparisons of mean scores between male and female drivers, offering a straightforward overview of group-level differences. Such results are useful for identifying broad patterns and highlighting where potential disparities may exist. In contrast, ordinal logistic regression estimates the adjusted effect of sex while simultaneously accounting for covariates such as age and driving experience, and it models the probability of responses across ordered categories rather than relying solely on mean values. This approach provides a more nuanced picture of how gender influences the likelihood of engaging in specific driving behaviours. Consequently, a behaviour that appears significant in the raw mean comparisons may lose significance after controlling for confounding variables, or the apparent direction of the effect may shift. For these reasons, both analyses were intentionally applied in a complementary manner. Descriptive statistics serve as an accessible and transparent first step, enabling comparisons with existing survey-based literature that commonly relies on similar methods. Ordinal logistic regression, on the other hand, strengthens the robustness of the findings by adjusting for individual characteristics and by modelling the data in a way that better reflects its structure. Together, the two approaches provide a more comprehensive and reliable assessment of sex differences in risky driving behaviours than either method would alone. To better understand the interaction between driving environment and gender, and their implications for traffic safety interventions, the results are discussed both in relation to sex and to road type.

### Differences related to sex

Sex differences were consistently observed across multiple behaviours. In line with previous researches [6,21,28,29], this study reveals that men are more prone to speeding across all driving conditions and road types, while women are more likely to drive below the speed limit, especially in high-stress situations such as traffic congestion. However, a distinctive finding of this study is that female drivers were more likely to report engaging in excessive speeding (over 30 km/h above the limit) on roads with higher permitted speeds (60–80 km/h). This extreme speeding tendency among women, that is reported with descriptive analysis and confirmed by the ordinal logistic regression, has not been emphasized in prior research. A possible explanation for this deviation could be that the analysis was based on self-reported behaviour and women may be more forthcoming in admitting risky behaviours, while men may underreport such violations. Also, factors such as the structure of the road network, levels of enforcement, and cultural perceptions of risk in this developing country may differ from those in developed countries, where most prior studies were conducted. Because all of the mentioned, this result should be interpreted with caution and can serve as a starting point for future research. In addition, men were more likely to report extreme speeding (more than 20 km/h over the limit) on roads with lower speed limits (50–60 km/h), indicating that the risk of extreme speeding may differs by road type.

Regarding driving under the influence of alcohol, while previous research [5] suggests that awareness of the risks associated with driving under the influence of alcohol is generally lower in developing countries, the findings from this study indicate that the majority of drivers do not engage in this behaviour. This suggests a higher level of risk awareness than what would typically be expected based on broader findings related to developing countries. Descriptive results indicate that men are more frequently engage in driving under the influence of alcohol, especially in urban areas, which is consistent with earlier studies [2,16,18,22]. However, while the t-test showed marginal statistical significance of gender differences in self-reported alcohol-impaired driving, the ordinal logistic regression did not confirm a strong gender effect. The negative coefficient in the regression model suggests that female drivers were somewhat more likely to self-report this behaviour, but the result was not statistically significant, indicating that gender alone is not a strong predictor of this behaviour. This finding diverges from previous research and may indicate the influence of cultural factors affecting self-reporting behaviour.

The conducted analysis shows differences in seatbelt usage. Descriptive data indicate that men more often report using a seatbelt on regional and urban roads, while the regression results show that the probability of wearing a seat belt is still higher in women. This last finding aligns with previous research showing that women are generally more compliant with seatbelt use across various traffic environments, while men are less likely to wear seatbelts or encourage their passengers to do so [16,23,26,38]. The study highlighted a significant difference, with the sex gap in seatbelt usage being most pronounced on urban and regional roads. The differences between the t-test and regression results stem from the fact that the regression model includes control variables (age and driving experience), while the t-test only compares mean values between sexes. Once these controls are introduced, part of the effect initially attributed to gender is explained by differences in age and experience. This provides a more accurate picture of the extent to which driving behaviour is truly associated with sex, independent of other influencing factors. In contrast, no significant sex differences were observed regarding the use of child restraint systems, suggesting that both men and women recognize the importance of child safety in vehicles. This finding implies that while sex -based behavioural disparities exist in personal safety measures (e.g., seatbelt usage), collective safety measures, such as child seat utilization, may be more universally accepted. Given that seatbelt compliance is a critical factor in reducing the severity of injuries in traffic crashes, policy measures should focus on increasing awareness and enforcement, particularly among male drivers.

The relationship between sex and mobile phone use while driving is complex and varies depending on the type of road and the specific phone-related behaviour. Previous research indicates that men are more likely to use mobile phones while driving and are generally more prone to distractions such as talking on the phone, smoking, and eating while driving [26,38], regardless of a country’s level of development. In contrast, while the t-test found that men were significantly more likely to report using hands-free devices on urban roads, it diverged from global findings by revealing no significant sex differences in texting or social media use. This suggests that both male and female drivers exhibit similar tendencies to engage in these behaviours across all road categories. Such deviation from established trends suggests that factors such as cultural, infrastructural, or enforcement-related influences may play a significant role in shaping driver behaviour in developing countries, highlighting the need for further investigation into contextual factors influencing this behaviour.

### Differences related to road category

The findings of this study confirm that different road categories significantly influence driver behaviour. Urban roads were associated with more cautious driving, whereas speeding violations were more frequent on main and regional roads.

On main roads, speeding behaviours differed moderately between male and female drivers. While both genders reported to exceeding speed limits, as mentioned earlier, particularly noteworthy finding relates to women who more frequently reported excessive speeding, particularly above 30 km/h, compared to males. Although this sex related issue stands in contrast to prior studies, which typically report men as more prone to extreme speeding, it aligns with existing research [9,10,32–34] indicating that main and regional roads present higher safety risks. A potential explanation are specific cultural perceptions of highway safety, lower levels of traffic enforcement in developing country or structural characteristics. Alcohol-impaired driving on main roads remained low for both sexes, however, the trend suggested slightly safer self-reported behaviour among men rather than women. Although women showed higher seatbelt compliance for themselves or for passengers on board, no significant sex differences were detected, suggesting that this behaviour may be less influenced by sex when road design inherently emphasizes safety. Finally, mobile phone use revealed clearer gender differences with males more likely to use hands-free devices, while texting and visiting social networks were equally common across sexes.

On regional roads, sex related differences became more pronounced. Females showed a slightly higher tendency toward extreme speeding (more than 20 km/h above the limit), although the differences were marginal. A significant positive coefficient for seatbelt usage, obtained with regression analysis, shows that women were more likely than men to wear seatbelts in this context, that aligns with previous research [16,38]. Regarding mobile phone use, men demonstrated greater use of hands-free devices, while use of a mobile phone while driving to write messages and visit social networks did not report significant difference.

Some differences in behaviour between the sexes in an urban environment can be discerned from the survey responses. Speeding violations were less frequent among women. Women reported safer behaviours for alcohol and seatbelt usage. Interestingly, when considering passenger seatbelt use, men reported slightly higher enforcement on local roads, but this effect was marginal. Also, the use of mobile phone for texting and visiting social media were more prevalent in urban contexts, likely due to slower traffic and more frequent stops at intersections.

### Psychological, cultural and infrastructural factors

A deeper theoretical elaboration of the findings suggests that the observed sex and road-type differences can be better understood through the interaction of psychological, cultural, and infrastructural factors. From a psychological perspective, theories of risk perception indicate that males generally exhibit higher sensation-seeking tendencies and greater tolerance for risk, while females are more compliant with traffic regulations and more sensitive to safety concerns [22,25,44–46]. These tendencies are also influenced by personality traits and driving experience, which mediate self-reported risky behaviours and accident risk [47,48]. This may explain why men in our sample more often reported extreme speeding on roads with lower limits, whereas women demonstrated a greater tendency for excessive speeding on high-speed roads, potentially perceiving such environments as safer due to road design and less frequent enforcement [9,33,49]. Culturally, sex roles in developing countries may reinforce these differences: risk-taking remains more socially acceptable among male drivers, while female drivers are expected to act more responsibly, which can also affect self-reporting behaviours [23,38]. Infrastructural and regulatory conditions further shape these outcomes. Limited enforcement, weaker safety campaigns, and design characteristics of regional and high-speed roads may create a false sense of security, amplifying risky behaviours in certain contexts [9]. Taken together, these findings suggest that differences observed in seatbelt usage (where women were more likely to comply when adjusting for age and driving experience) and speeding patterns cannot be fully explained by sex alone, but are embedded in broader psychological dispositions, cultural expectations, and infrastructural environments typical of developing countries [38].

Overall, these results confirm that gender and road type shape general patterns of driving behaviour, underlining the need for targeted interventions adapted to specific road environments.

## Conclusion

This study examined sex differences in risky driving behaviours across various road categories in a developing country context. The findings show that male drivers generally demonstrate riskier behaviour related to speeding, alcohol use, and seatbelt compliance. However, an important and unexpected result was that female drivers reported higher rates of excessive speeding on high-speed roads, which contrasts with existing evidence from developed countries. These results highlight that both sex and road type interact in shaping unsafe behaviours, and that contextual factors in developing countries must be considered when designing road safety measures. The study contributes to the literature by extending knowledge on sex differences in traffic safety to a developing country setting, where empirical evidence is still scarce. In particular, it identifies patterns of behaviour that differ from global trends, such as woman’s self-reported engagement in extreme speeding on highways, thus underlining the importance of local cultural, infrastructural, and enforcement conditions in shaping driver behaviour. The findings of this study highlight the need for tailored traffic interventions that address sex-specific risk behaviours. Stricter enforcement on urban roads and targeted campaigns addressing alcohol-impaired driving may be particularly effective among men, while awareness programmes on the dangers of excessive speeding should be prioritised for female drivers on high-speed roads. Additionally, interventions should extend beyond sex-specific messaging to address broader factors influencing alcohol-impaired driving, such as cultural attitudes toward drinking and driving and the availability of alternative transportation options. Efforts should focus on stricter enforcement measures and public safety campaigns that highlight the risks of non-compliance with seatbelt usage, particularly among men, as these strategies may be effective in addressing this issue. However, the lack of significant sex differences in child restraint system usage suggests that collective safety measures are widely accepted, emphasizing the role of cultural norms in shaping responsible driving behaviours. The observed relationship between driver sex and patterns of mobile phone use underscores the need for broader safety policies that mitigate all distractions, moving beyond sex-specific behavioral trends. Public awareness initiatives should highlight the dangers of both handheld phone use and texting while driving, particularly in urban environments, where distractions can have severe consequences.

Several limitations of this study need to be addressed. One of the main concerns is the data collection method. In most developing countries, behavioural data are typically gathered through surveys, which have several inherent limitations. First, the answers are based on the respondents’ personal perceptions, which can result in inaccurate or unreliable data. Next, responses may be affected by social desirability bias, where individuals may report behaviours that align with societal expectations rather than their true actions. While steps were taken to minimize this bias, such as ensuring anonymity during the data collection process (as recommended in [50]), it remains a significant limitation of the current study. Another issue is the potential subjective interpretations of the Likert scale responses, as different respondents may understand terms like “usually” differently, and the perceived distance between scale points might not be consistent across participants. In addition, although the present analysis combined descriptive tests and ordinal logistic regression, offering a broader perspective on driving behaviours, the fact that these methods occasionally lead to different conclusions indicate that sex effects are partly shaped by age and driving experience and also underlines the need for additional analytical approaches and robustness checks. Furthermore, self-reported data, while valuable, are inherently subjective and may not fully capture actual driving behaviours. So, future studies should incorporate objective data sources, such as GPS tracking, speed sensors, or in-vehicle telematics, to validate or challenge the current findings, particularly in cases that contradict prior research such as that female drivers are more likely to engage in extreme speeding on high-speed roads.

Additionally, future research should adopt an interdisciplinary approach that integrates psychological, cultural, and behavioural frameworks to explain the identified sex differences in driving behaviour. Importantly, longitudinal designs should be employed to examine potential changes in driving behaviours over time, enabling researchers to determine whether sex-related patterns are stable or evolve across different life stages and driving experiences. A deeper understanding of these underlying mechanisms could provide a stronger theoretical basis for the findings and support the development of more effective, evidence-based interventions and policy measures.

Overall, this study underscores the importance of analysing sex-specific driving behaviours in relation to road type within developing country contexts. By recognising these patterns, policymakers can design evidence-based interventions that address risks related to both sex and road type, thereby improving traffic safety and reducing crash rates.

### Declaration of generative AI and AI-assisted technologies in the writing process

During the preparation of this work the authors used https://chatgpt.com/ in order to insure grammatically correct use of English language. After using this tool/service, the authors reviewed and edited the content as needed and take full responsibility for the content of the publication.
